# Divalent Cations Regulate the Ion Conductance Properties of Diverse Classes of Aquaporins

**DOI:** 10.3390/ijms18112323

**Published:** 2017-11-03

**Authors:** Mohamad Kourghi, Saeed Nourmohammadi, Jinxin V. Pei, Jiaen Qiu, Samantha McGaughey, Stephen D. Tyerman, Caitlin S. Byrt, Andrea J. Yool

**Affiliations:** 1 Adelaide Medical School, University of Adelaide, Adelaide, SA 5005, Australia; mohamad.kourghi@adelaide.edu.au (M.K.); saeed.nourmohammadi@adelaide.edu.au (S.N.); jinxin.pei@adelaide.edu.au (J.V.P.); 2 School of Agriculture, Food and Wine, University of Adelaide, Adelaide, SA 5005, Australia; jiaen.qiu@adelaide.edu.au (J.Q.); samantha.mcgaughey@adelaide.edu.au (S.M.); steve.tyerman@adelaide.edu.au (S.D.T.); caitlin.byrt@adelaide.edu.au (C.S.B.)

**Keywords:** water channel, aquaporin, *Arabidopsis*, *Drosophila*, vertebrates, invertebrates, regulation, divalent cations

## Abstract

Aquaporins (AQPs) are known to facilitate water and solute fluxes across barrier membranes. An increasing number of AQPs are being found to serve as ion channels. Ion and water permeability of selected plant and animal AQPs (plant *Arabidopsis thaliana* AtPIP2;1, AtPIP2;2, AtPIP2;7, human *Homo sapiens* HsAQP1, rat *Rattus norvegicus* RnAQP4, RnAQP5, and fly *Drosophila* *melanogaster* DmBIB) were expressed in *Xenopus* oocytes and examined in chelator-buffered salines to evaluate the effects of divalent cations (Ca^2+^, Mg^2+^, Ba^2+^ and Cd^2+^) on ionic conductances. AtPIP2;1, AtPIP2;2, HsAQP1 and DmBIB expressing oocytes had ionic conductances, and showed differential sensitivity to block by external Ca^2+^. The order of potency of inhibition by Ca^2+^ was AtPIP2;2 > AtPIP2;1 > DmBIB > HsAQP1. Blockage of the AQP cation channels by Ba^2+^ and Cd^2+^ caused voltage-sensitive outward rectification. The channels with the highest sensitivity to Ca^2+^ (AtPIP2;1 and AtPIP2;2) showed a distinctive relief of the Ca^2+^ block by co-application of excess Ba^2+^, suggesting that divalent ions act at the same site. Recognizing the regulatory role of divalent cations may enable the discovery of other classes of AQP ion channels, and facilitate the development of tools for modulating AQP ion channels. Modulators of AQPs have potential value for diverse applications including improving salinity tolerance in plants, controlling vector-borne diseases, and intervening in serious clinical conditions involving AQPs, such as cancer metastasis, cardiovascular or renal dysfunction.

## 1. Introduction

Maintaining water homeostasis in living organisms is fundamental for survival. Aquaporins (AQPs) in the Major Intrinsic Protein (MIP) family facilitate the transport of water and other solutes across biological membranes [1], including urea, glycerol, nonpolar gases, hydrogen peroxide, and metalloids [2]. AQPs are expressed in bacteria, protists, plants, invertebrates and vertebrates [3]. Ion channel function has been demonstrated for a subset of members of the MIP family, with examples including both anion- and cation-selective channels. Anion-selective channels characterized to date include mammalian AQP0 (lens MIP), which is regulated by pH [4,5,6] and essential for maintaining the optical clarity of the lens [7], and mammalian AQP6, associated with intracellular vesicles and activated by low pH [8].

Cation-selective aquaporin ion channels include mammalian AQP1 gated by cyclic guanosine monophosphate (cGMP) [9,10], *Drosophila* big brain (DmBIB) modulated by tyrosine phosphorylation [11,12], and *Arabidopsis* Plant membrane Intrinsic Protein PIP2;1 sensitive to pH and calcium [13]. AQP cation channels have been linked to diverse functional roles. Mammalian AQP1 expression has been associated with migration and metastasis in aggressive cancers including colon, melanoma and breast cancers, astrocytoma and glioblastoma [14,15,16,17,18]. An arylsulfonamide inhibitor of the AQP1 ion channel, AqB011, significantly impaired migration in the AQP1-expressing colon cancer cell line HT29 [19]. The AQP1 and AQP4 water channel blocker AqB013 also slowed cancer cell migration [20,21], suggesting that both AQP1 water and ion channel functions are involved in facilitating cell mobility. Two medicinal plant components, bacopaside I and bacopaside II from *Bacopa monnieri,* blocked AQP1 water channels and also inhibited the migration of AQP1-expressing colon cancer cells [22]. *Drosophila* Big Brain (BIB) is important in the early development of the fly nervous system [23]; inherited mutations in *bib*, one of the neurogenic genes, result in impaired lateral inhibition and the overproduction of neuroblasts [24]. The model plant *Arabidopsis thaliana* has 35 AQP isoforms [25], with the plasma membrane intrinsic proteins AtPIP2;1, AtPIP2;2 and AtPIP2;7 being among the most highly expressed in the roots [26]. All three aquaporins are important for the regulation of water flow through plants and AtPIP2;1 is also implicated in signaling for stomatal closure [27,28]. Both AtPIP2;1 and AtPIP2;7 are regulated by salt stress [29,30] making the modulation of different AQPs by divalent cations an important consideration in understanding function.

Direct modulation of many types of ion channels and receptors by divalent cations is a ubiquitous component of cellular signal transduction and regulatory transport mechanisms [31,32,33,34,35,36]. Voltage-sensitive relief of Mg^2+^ block of excitatory NMDA (*N*-methyl-d-aspartate) receptors, for example, is essential for the establishment of neuronal long-term potentiation [37]. Voltage-sensitive unblocking of Mg^2+^ in inwardly rectifying K_ir_ potassium channels enables differential control of repolarization as a function of membrane potential in diverse types of excitable cells [38]. Many classes of K^+^, Ca^2+^, Cl^−^ and Na^+^ channels are activated by intracellular calcium or Ca^2+^-dependent kinases that govern solute transport across membranes, baseline excitability and action potential duration and frequency.

Accumulating evidence suggests that AQP channels from across phyla are similarly modulated by divalent cations. Prior work showed the mammalian AQP1 ion conductance is blocked by Cd^2+^ [39], the insect BIB channel shows voltage-sensitive block by Ca^2+^ and Ba^2+^ [12], and both water and ion channel activities of *Arabidopsis* PIP2;1 are blocked by Ca^2+^ [13,40]. Discovering the Ca^2+^ sensitivity was the key step needed for uncovering the ionic conductance property of the AtPIP2;1 channel [13], identifying a molecular mechanism for an undefined cationic current previously associated with root adaptation responses to environmental factors [41]. 

Work here compares the dose-dependent blocking effects of divalent cations across a diverse array of AQP channels. As well, results here are the first to define a new AQP ion channel from *Arabidopsis*, AtPIP2;2, with high sensitivity to inhibition by Ca^2+^. In summary, our comparative analysis shows that the block of AQP ion channels by divalent cations is a regulatory mechanism that is common across diverse classes of AQP channels. Understanding the mechanisms that regulate AQP ion channel function is essential for the continuing discovery of new members of AQP ion channel group, and for defining the diverse physiological roles and value of AQPs as targets for intervention in health care, disease vector management, and agricultural translational applications.

## 2. Results

### 2.1. Expression of Aquaporin (AQP) Channels in Xenopus Oocytes

**Figure 1:**
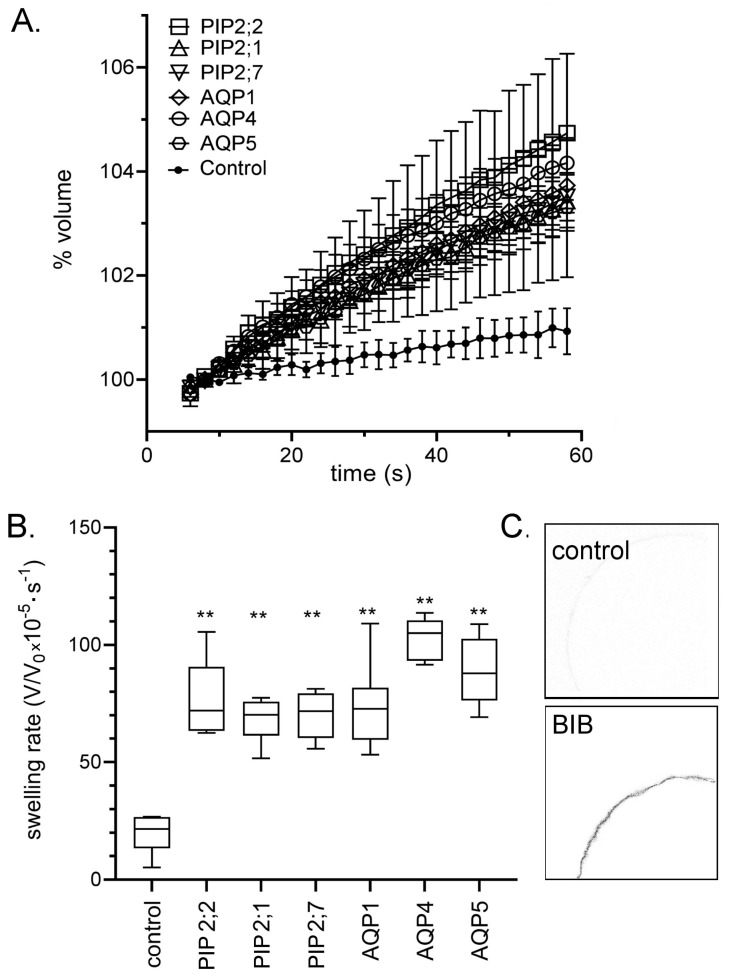
Confirmation of the expression of AQP1, AQP4, AQP5, PIP2;7, PIP2;2, PIP2;1, and BIB channels in *Xenopus* oocyte membranes. (**A**) Osmotic swelling responses as a function of time in 50% hypotonic saline for aquaporins (AQP)-expressing oocytes as compared with non-AQP-expressing control oocytes. Data are mean ± SEM; *n* = 6 per treatment group. (**B**) Box plots of swelling rates for the data shown in (**A**). ANOVA and post hoc Bonferroni test; ** (*p* < 0.01) as compared with control; *n* = 6 per group. (**C**) Confocal image of immuno-labeled oocytes confirming BIB protein expression in the oocyte plasma membrane, as described previously [11].

To confirm successful heterologous expression of the different classes of AQP channels in *Xenopus* oocytes, swelling assays in 50% hypotonic saline were performed for AtPIP2;7, AtPIP2;2, AtPIP2;1, RnAQP4, RnAQP5, and HsAQP1 copy-RNA-injected oocytes (Figure 1). The osmotic water permeability was significantly greater in oocytes expressing AtPIP2;7, AtPIP2;2, AtPIP2;1, RnAQP4, RnAQP5, or HsAQP1 than in non-AQP-expressing control oocytes, confirming the expression of functional channels in the oocyte membrane (Figure 1A). Compiled data for osmotic water permeabilities are summarized in a box plot (Figure 1B). DmBIB channels do not show appreciable osmotic water permeability [11]; thus the expression of DmBIB channels on oocyte plasma membranes was confirmed by immunostaining and confocal microscopy (Figure 1C). The DmBIB channels used here are hemagglutinin-epitope-tagged BIB, which is the same construct that was referred to as HA-BIB in the work by Yanochko and colleagues previously [11].

### 2.2. Differential Sensitivity of AQP Ion Currents to Block by Ca^2+^

**Figure 2:**
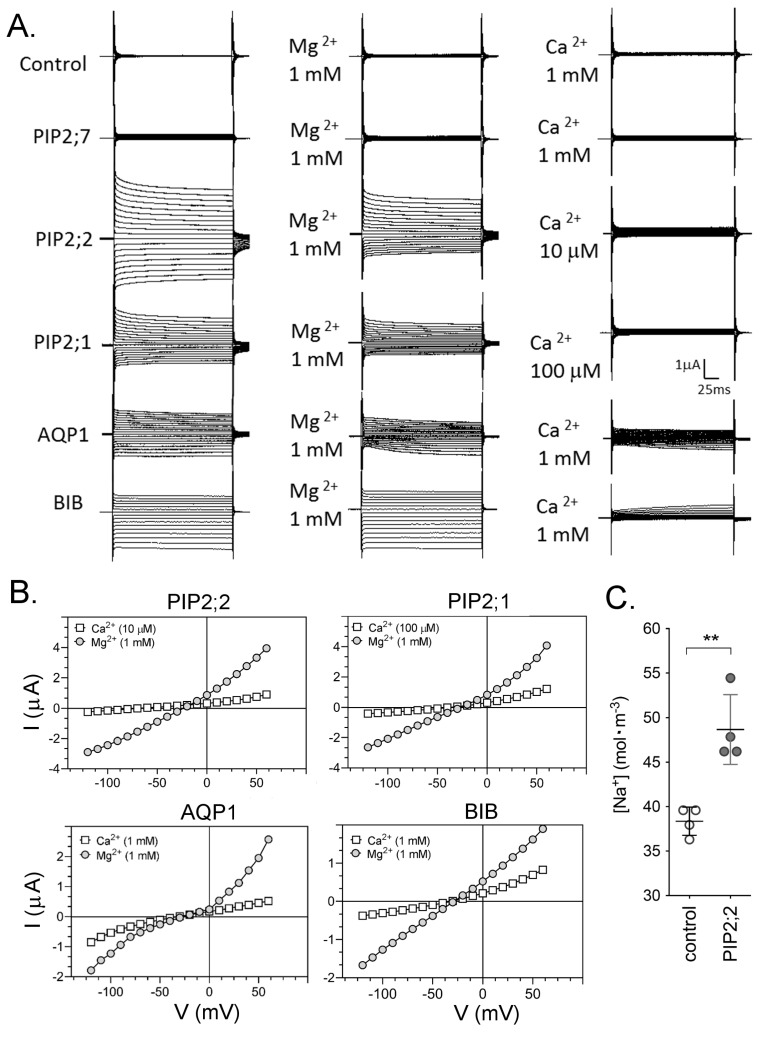

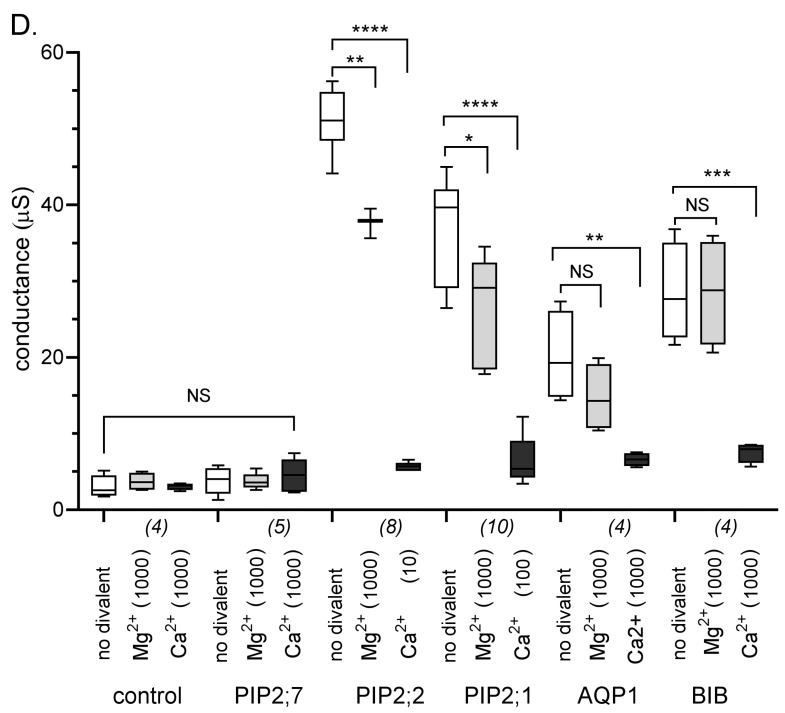
Effects of Mg^2+^ and Ca^2+^ divalent cations on ionic current responses in oocytes expressing different classes of AQPs. (**A**) Superimposed currents as a function of time measured by voltage clamp (steps from −110 to +60 mV, from a holding potential of −40 mV) at maximal activation in divalent-free saline (**left**), after application of Mg^2+^ (**middle**), and after application of Ca^2+^ (**right**). Control and PIP2;7-expressing oocytes lacked appreciable conductances. (**B**) Current-voltage relationships for the traces illustrated in (**A**). (**C**) Na^+^ concentrations in oocytes expressing PIP2;2 as compared with control oocytes after incubation in Frog Ringers containing 96 mM NaCl for three days. Data are from four replicates; each replicate contained five oocytes. (**D**) Box plot summary of compiled data for the conductances. Free Ca^2+^ concentrations are given in μM. **** (*p* < 0.0001); *** (*p* < 0.001); ** (*p* < 0.01); * (*p* < 0.05); NS (not significant); using ANOVA with *post-hoc* Bonferroni tests. *n* values are in italics below the *x*-axis.

AQP ion channel currents were measured in oocytes using a voltage clamp (Figure 2). HsAQP1-expressing oocytes were activated by bath application of a membrane-permeable cyclic GMP analog at 10 μM (see Methods), as per established protocols [9]. Ionic conductances in AtPIP2;1 [13], AtPIP2;2 and DmBIB [11] expressing oocytes were evident when the recording electrodes were inserted into the oocytes, suggesting that, in the batches of oocytes tested, these channels were already active in the expression system (Figure 2A). In contrast, non-AQP-expressing control oocytes and oocytes expressing AtPIP2;7 showed no ionic conductance responses during 30-min recording sessions. 

As shown in Figure 2A, current traces were recorded for control and AQP-expressing oocytes after maximal activation in divalent-free saline (left); after perfusion of bath saline with Mg^2+^ (middle); and after washout and application of EGTA-buffered Ca^2+^ saline for the same oocytes (right). AtPIP2;2 and AtPIP2;1 currents were reduced slightly by Mg^2+^ at 1 mM. All four classes of AQP ion channels were sensitive to Ca^2+^. In AtPIP2;2-expressing oocytes, 10 μM calculated free Ca^2+^ conferred maximum inhibition. In oocytes expressing AtPIP2;1 maximum inhibition was observed at 100 μM free Ca^2+^. HsAQP1-expressing oocytes showed little effect of Mg^2+^, and moderate inhibition by Ca^2+^ at 1 mM. Similarly, the ion conductance in DmBIB-expressing oocytes was not blocked by Mg^2+^; and was inhibited by 1 mM extracellular Ca^2+^. 

Current-voltage relationships for traces in panel A are shown in Figure 2B. Current-voltage relationships were approximately linear in Ca^2+^ and Mg^2+^ salines. Measurement of the accumulation of Na^+^ in control and AtPIP2;2-expressing oocytes after three days in Frog Ringers saline (containing 96 mM NaCl) indicated that AtPIP2;2 expression was associated with an increased oocyte Na^+^ content (Figure 2C). Compiled conductance data are summarized in a box plot (Figure 2D). The ion conducting AQP channels showed block by Ca^2+^, with an order of sensitivity PIP2;2 > PIP2;1 > BIB > AQP1. The low baseline ionic conductance levels seen for non-AQP-expressing control oocytes and oocytes expressing AtPIP2;7 were not substantially altered by Ca^2+^ or Mg^2+^ application. 

### 2.3. Voltage-Sensitive Block of AQP Ion Channels by Ba^2+^

**Figure 3:**
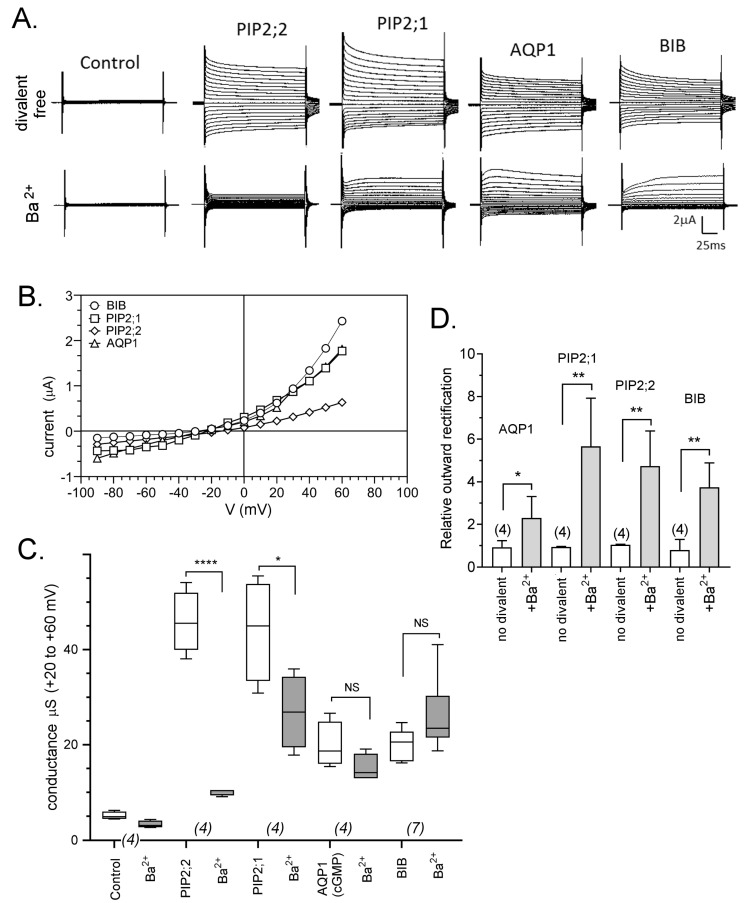
Effects of Ba^2+^ on ionic currents in oocytes expressing different AQPs. (**A**) Superimposed current traces as a function of time measured by voltage clamp at maximal activation in divalent-free saline (**upper** row) and after perfusion with bath saline containing 1 mM BaCl_2_ (**lower**). (**B**) Current-voltage relationships in Ba^2+^ for traces presented in (**A**). (**C**) Box plot summary of compiled data for AQP1, PIP2;1, PIP2;2 and BIB channels before and after 1 mM Ba^2+^ application. (**D**) Histogram showing the relative outward rectification values (I_+60_/I_−80_) as mean ± SEM. Ratios were calculated as amplitude of outward (+60 mV) to inward (−80 mV) currents. **** (*p* < 0.0001); ** (*p* < 0.001); * (*p* < 0.01); NS (not significant); using ANOVA with post-hoc Bonferroni tests. *n* values are near the *x*-axis.

BaCl_2_ (1 mM) applied extracellularly induced outward rectification of the ion currents recorded in oocytes expressing AtPIP2;1, AtPIP2;2, HsAQP1 or DmBIB channels (Figure 3). After Ba^2+^ application, the AtPIP2;1 and AtPIP2;2 ion channels showed an overall decrease in current amplitude consistent with inhibition, as well as a change in the kinetics of the outward current that was consistent with a process of time- and voltage-sensitive unblocking (Figure 3A). Currents recorded for the HsAQP1 and DmBIB expressing oocytes showed comparable unblocking of the outward currents, but the unblocked currents reached peak amplitudes that were not significantly different from the corresponding initial currents measured without Ba^2+^. 

Figure 3B illustrates current-voltage relationships in Ba^2+^ for traces shown in Figure 3A. A summary of the conductance values for outward currents (measured before and after Ba^2+^ application) is shown in the box plot (Figure 3C). Figure 3D shows relative outward rectification ratios (I_+60_/I_−80_*)* for HsAQP1, DmBIB, AtPIP2;1 and AtPIP2;2, confirming significant outward rectification of currents in saline with 1 mM Ba^2+^.

### 2.4. Inhibition of AQP Ion Channels by Cd^2+^

**Figure 4:**
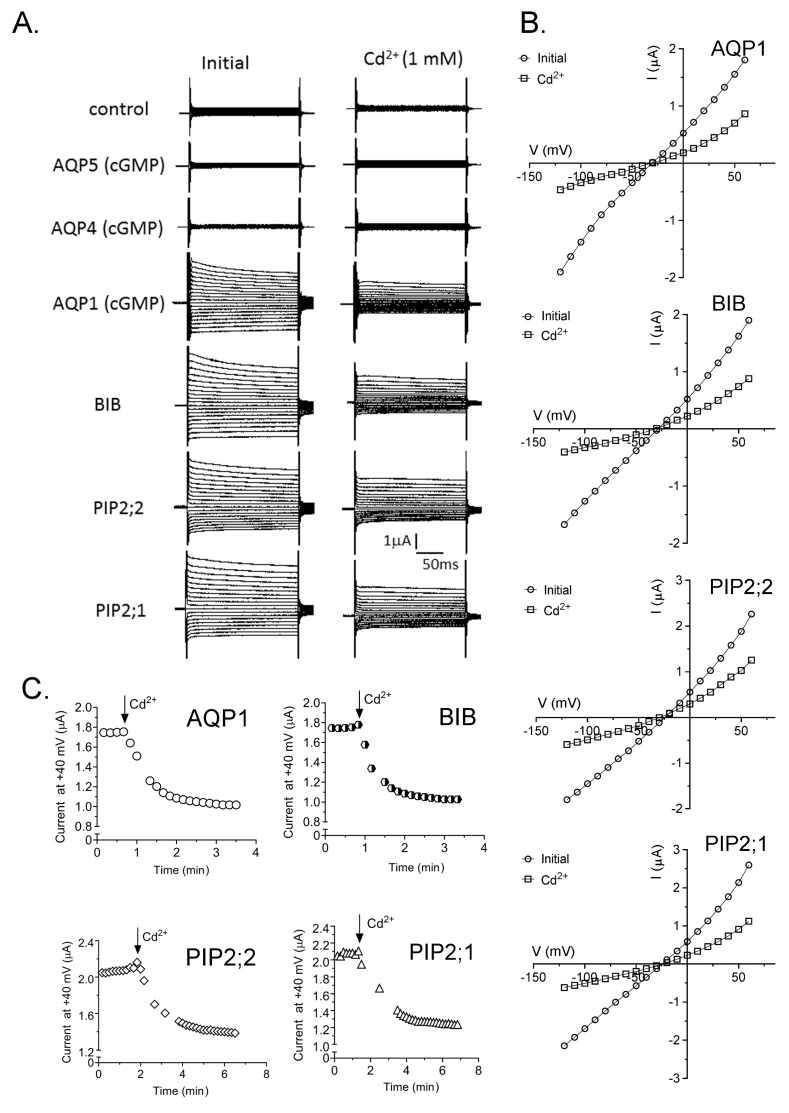

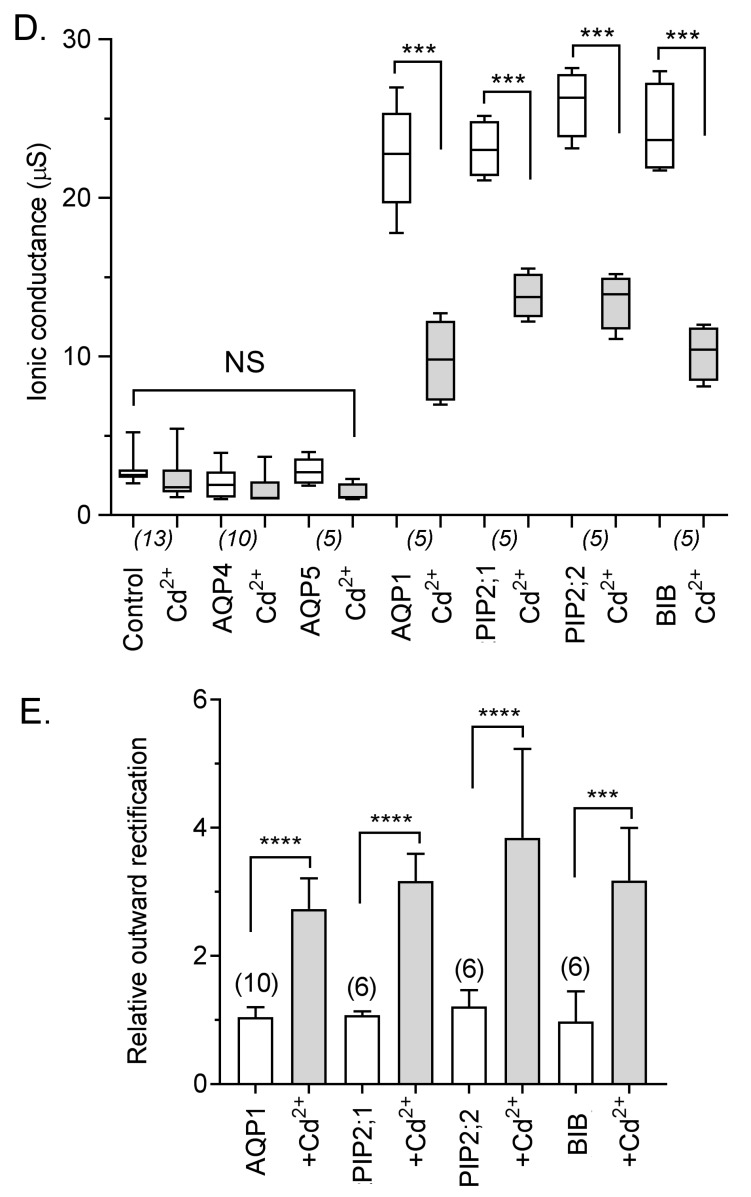
Effects of Cd^2+^ on ion current responses in oocytes expressing different classes of AQPs. (**A**) Superimposed current traces under voltage clamp recorded at maximal activation (if present) in divalent-free saline (**left**) and after perfusion with bath saline containing 1 mM CdCl_2_ (**right**). (**B**) Current-voltage relationships for data presented in (**A**). (**C**) Rates of onset of block after perfusion of bath saline containing 1 mM Cd^2+^, as measured using repeated voltage steps to +40 mV. (**D**) Box plot summary of compiled data for control, AQP4, AQP5, AQP1, PIP2;1, PIP2;2 and BIB expressing oocytes before and after Cd^2+^ application. *n* values are below the *x*-axis. (**E**) Histogram showing relative outward rectification values (mean ± SEM), calculated as the ratio of outward to inward currents at +60 and −80 mV (I_+60_/I_-80_). **** (*p* < 0.0001); *** (*p* < 0.005); NS (not significant); using ANOVA with post-hoc Bonferroni tests; *n* values are shown above the histogram bars.

Ion conducting AQPs were blocked by extracellular application of Cd^2+^ (Figure 4). After establishing maximal ionic conductance responses in divalent-free saline (initial), perfusion with bath saline containing 1 mM CdCl_2_ resulted in inhibition of the ion currents in HsAQP1, DmBIB, AtPIP2;1 and AtPIP2;2 expressing oocytes (Figure 4A). Mammalian AQP4 and AQP5 failed to show any ionic conductance responses, with or without application of CPT-cGMP, and showed no effect of Cd^2+^, remaining comparable to non-AQP-expressing control oocytes. Figure 4B illustrates the current-voltage relationships for traces in panel A. Figure 4C shows the rate of onset of inhibition (within minutes) after application of saline with 1 mM Cd^2+^. Figure 4D shows compiled data in a box plot summarizing the effects of 1 mM CdCl_2_ on conductances recorded in HsAQP1, DmBIB, AtPIP2;1 and AtPIP2;2 expressing oocytes. Figure 4E illustrates the relative outward rectification values (I_+60_/I_-80_) measured in the absence and presence of extracellular Cd^2+^, with ratio values increased 3- to 4-fold in Cd^2+^ saline. 

### 2.5. Relief of Ca^2+^ Inhibition by Addition of Ba^2+^

**Figure 5:**
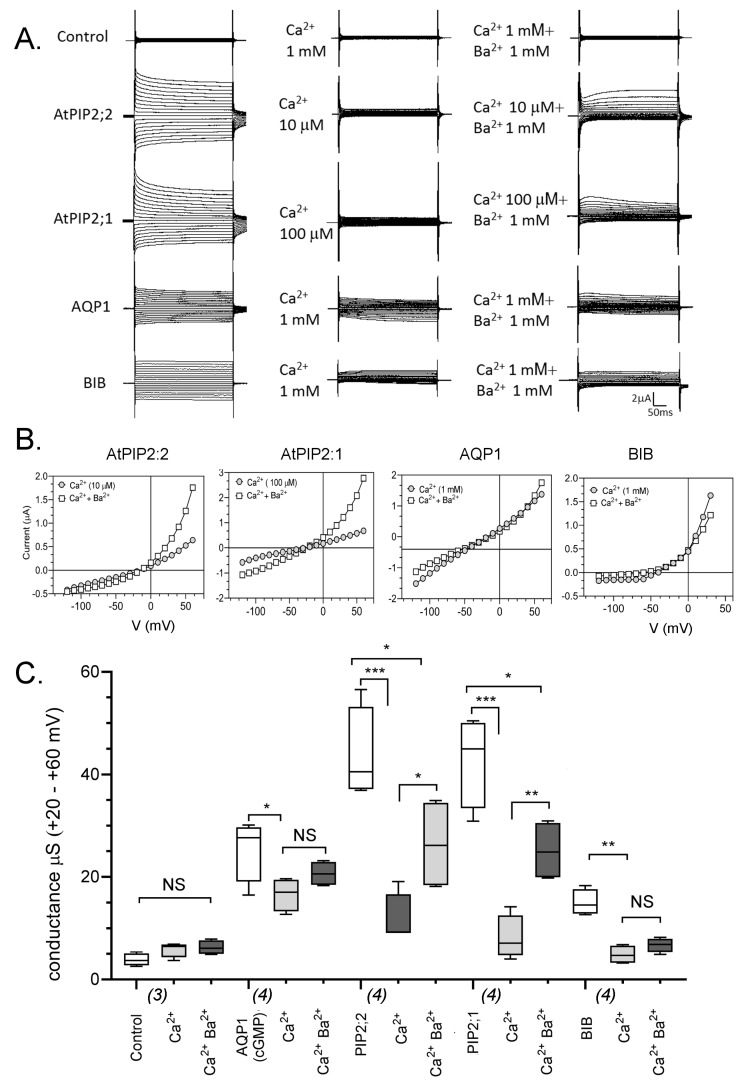
Effects of sequential application of Ca^2+^ followed by Ba^2+^ on ionic current responses in oocytes expressing different classes of AQPs. (**A**) Superimposed current traces under voltage clamp showing the maximal responses of AtPIP2;2, AtPIP2;1, HsAQP1 and DmBIB expressing oocytes recorded in divalent-free saline (**left**), after perfusion of saline containing the indicated amount of free Ca^2+^ (**middle**), and after application of Ba^2+^ in the continuing presence of the same concentration of Ca^2+^ (**right**). (**B**) Current-voltage relationships for traces shown in (**A**). (**C**) Summary box plot of conductance values for AtPIP2;2, AtPIP2;1, HsAQP1 and DmBIB expressing oocytes in different divalent cation salines. *** (*p* < 0.001); ** (*p* < 0.01); * (*p* < 0.05); NS (not significant); using ANOVA with post hoc Bonferroni tests; *n* values are below the *x*-axis.

Cumulative application of Ca^2+^ followed by Ba^2+^ was used to investigate possible competitive interactions between divalent cation blockers (Figure 5). After recording initial activated responses in Ca^2+^ free saline (Figure 5A, left), salines containing the optimal concentrations of free Ca^2+^ needed for inhibition were perfused into the recording chamber. Final concentrations of 1 mM CaCl_2_ were used for HsAQP1 and DmBIB-expressing oocytes, 100 μM was used for AtPIP2;1 and 10 μM for AtPIP2;2 expressing oocytes, and responses were recorded (Figure 5A, middle). 

Subsequent application of 1 mM BaCl_2_ in the continuing presence of the same concentration of CaCl_2_ resulting in a distinctive voltage-sensitive recovery of outward currents in AtPIP2;1 and AtPIP2;2 expressing oocytes (Figure 5A, right). Similar recovery was not evident in HsAQP1 and DmBIB expressing oocytes (but Ba^2+^ was not present in 10- to 100-fold excess for DmBIB and HsAQP1, as it was for the AtPIP channels). Figure 5B illustrates the current-voltage relationship for traces shown in Figure 5A. Results are summarized in a box plot (Figure 5C). 

### 2.6. Pharmacological Effects of the AQP Ion Channel Blocker, AqB011

**Figure 6:**
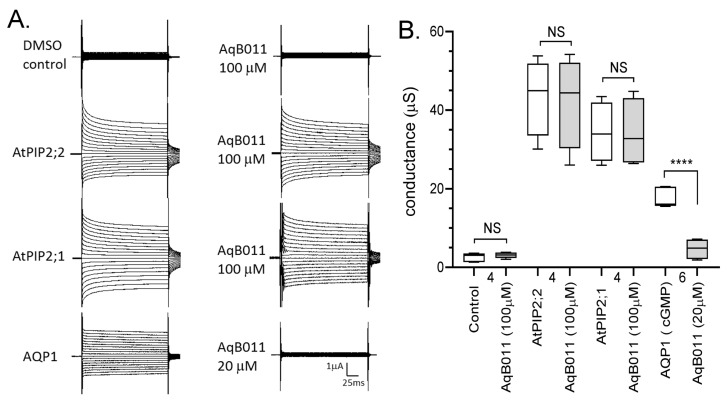
Effects of the arylsulfonamide compound AqB011 on ionic current responses in oocytes expressing different classes of AQPs. (**A**) Responses were recorded for control (DMSO vehicle), and AtPIP2;1, AtPIP2;2, and HsAQP1 expressing oocytes before (**left**) and after 2 h incubation (**right**) in AqB011 at the indicated doses. HsAQP1 was blocked by 20 μM AqB011; AtPIP2;1 and AtPIP2;2 currents were not affected by 100 μM AqB011. (**B**) Box plot summary of the conductance levels before and after treatment with AqB011. **** (*p* < 0.0001); NS (not significant); using ANOVA with post hoc Bonferroni tests; *n* values are below the *x*-axis.

The arylsulfonamide AqB011 was previously shown to inhibit human AQP1 ion channels [19]. Given the overlap in sensitivity to inhibition by divalent cations reported here for diverse classes of cation-selective AQPs, the possible use of AqB011 as a blocker of multiple classes of AQP cation-selective channels was evaluated (Figure 6). Oocytes expressing HsAQP1 were activated by CPT-cGMP to establish an initial responses, and then incubated for 2 h in divalent-free saline with or without 20 μM AqB011 and re-tested for activation by CPT-cGMP, following standard protocols [19]. Results confirmed the expected inhibition of the HsAQP1 ion channel by AqB011 (Figure 6A). AtPIP2;1 and AtPIP2;2 expressing oocytes were recorded in initial normal saline, then incubated for 2 h with 100 μM AqB011, and recorded again. In contrast to HsAQP1, no effect of AqB011 treatment was observed for AtPIP2;1 and AtPIP2;2 expressing oocytes. There were no differences in ionic conductance observed for oocytes expressing AtPIP2;1 and AtPIP2;2 with or without 100 μM AqB011 treatment, indicating that among the channels tested, the agent shows selectivity for the mammalian AQP1 ion channel. Non-AQP-expressing control oocytes treated with vehicle dimethylsulfoxide (DMSO) showed minimal baseline conductance, and no effect of incubation with AqB011 (2 h, 100 μM). Data for conductance values with and without AqB011 treatment are summarized in a box plot (Figure 6B). 

## 3. Discussion

Only a subset of AQPs is at present known to have ion channel function. Fifteen mammalian aquaporin genes have been identified (AQP0–AQP14) [42,43], three of which are ion channels (AQPs 0, 1 and 6). Ionic conductances have not been observed in oocytes expressing mammalian AQP4 or AQP5. HsAQP1 is thought to be activated via cGMP interaction with arginine residues in the loop D domain, and influenced by tyrosine phosphorylation in the carboxyl terminus [9,10,44]. Plants have a substantial number of AQP loci, divided into five subfamilies. One of the model plants, *Arabidopsis,* for example has 35 aquaporin genes [25]. The plant subfamily of Plasma membrane Intrinsic Proteins (PIPs) is divided into two groups, PIP1 and PIP2, with five and eight members, respectively. Results here are the first to show that the plant AQP AtPIP2;2 from *Arabidopsis* conducts an ion current when expressed in *Xenopus* oocytes in low Ca^2+^ saline. An ionic conductance was not observed for AtPIP2;7 expressing oocytes in the same conditions. AtPIP2;1 [13] and the soybean Nodulin 26 [45] also have ion channel activity. 

Osmotic water permeability assays or confocal microscopy confirmed that the diverse classes of AQPs (AtPIP2;7, AtPIP2;2, AtPIP2;1, RnAQP4, RnAQP5, HsAQP1 and DmBIB) were expressed and trafficked to the oocyte plasma membrane (Figure 1). Two electrode voltage clamp assays demonstrated the ion conducting properties of AtPIP2;2, AtPIP2;1, HsAQP1 and DmBIB expressing oocytes (Figure 2), which were distinct from control, and from oocytes expressing AtPIP2;7, RnAQP4 or RnAQP5 channels. Previously we demonstrated that AtPIP2;1 expression in oocytes enhanced the transport of Na^+^, as quantified by measuring internal Na^+^ accumulation and by measuring Na^+^ fluxes with external ion sensitive electrodes; these findings were corroborated by results with AtPIP2;1 expressing yeast, which also showed increased intracellular Na^+^ [13]. Here we report that AtPIP2;2 channels also enhanced Na^+^ transport, as measured by increased accumulation of Na^+^ in AtPIP2;2 expressing oocytes relative to control (Figure 2C). Low baseline currents recorded in non-AQP expressing control oocytes were similar to those in RnAQP4, RnAQP5, and AtPIP2;7 expressing oocytes, indicating these AQPs are unlikely to function as ion channels under the conditions tested. 

The lack of ionic conductance responses in oocytes expressing RnAQP4, RnAQP5 and AtPIP2;7 provide a valuable benchmark. These results indicate that it is unlikely that the ionic conductances observed for AtPIP2;2, AtPIP2;1, HsAQP1 and DmBIB were simply due to indirect mechanisms (such as activation of native oocyte channels by heterologous protein trafficking, osmotic water stress, or removal of extracellular divalent cations during recording sessions), since all of these parameters would have been replicated in oocytes expressing the non-ion conducting AQPs; however, no currents above baseline were observed for RnAQP4, RnAQP5 or AtPIP2;7. Possible contributions of the native oocyte Cl^−^ current activated by intracellular Ca^2+^ [46,47] were minimized by using a holding potential of −40 mV for the voltage-clamp recordings, which results in inactivation of the native voltage-gated Ca^2+^ channels, in accord with our standard practice for experiments on AQP ion channels. 

**Figure 7:**
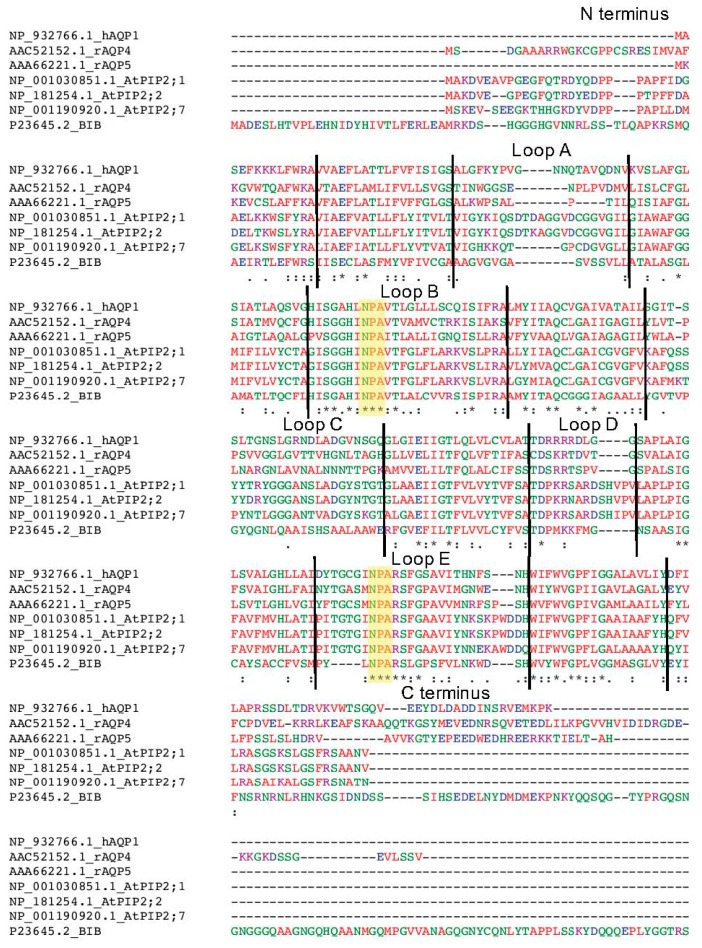
Amino acid sequence alignment of HsAQP1, RnAQP4, RnAQP5, AtPIP2;1, AtPIP2;2, AtPIP2;7 and DmBIB. Sequences from the NCBI Protein Database were aligned using Clustal Omega software (Available online: http://www.ebi.ac.uk/Tools/msa/clustalo/; accessed on 18 October 2017). HsAQP1 protein topology was from the Topology Data Bank of Transmembrane Proteins (TOPDB). Symbols: asterisk (*) identical residues across all sequences; colon (:) highly conserved residue; period (.) semi-conserved residue. Hyphens (-) show gaps; black vertical lines separate predicted domains, as labelled. Colors illustrate chemical properties: positive charged (magenta), negative charged (blue), polar (green), hydrophobic (red). Highlighted in yellow are NPA (Asn-Pro-Ala) signature motifs located in loops B and E, conserved in most aquaporins. The carboxy terminus of DmBIB was truncated for this figure.

Further research is needed to define the specific interaction sites for divalent cations as blockers of AQP channels. Pairs of negatively charged amino acids are characteristic of calcium binding sites in other channels [48]. Alignments of amino acid sequences (as illustrated in Figure 7) can offer testable predictions of candidate sites for further evaluation. Results here showed that application of Ca^2+^ inhibited the HsAQP1, DmBIB, AtPIP2;1 and AtPIP2;2 ionic conductances, with AtPIP2;1 and AtPIP2;2 channels showing the highest sensitivity, as well as relief of inhibition by the co-application of excess Ba^2+^. A number of possible sites have been implicated in AQP interactions with divalent cations, associated with both intracellular and extracellular domains of AQP channels. For example, wild type DmBIB is not blocked by Mg^2+^; however, mutation of glutamate (E^71^) to aspartate created a gain-of-function sensitivity to inhibition by Mg^2+^ [12]. The E^71^ residue is modeled from homologous crystal structures as being in the cytoplasmic half of the first transmembrane domain (M1). Data here showed that HsAQP1, DmBIB, AtPIP2;1 and AtPIP2;2 currents were blocked by Cd^2+^. In the spinach *(Spinacia oleracea*) SoPIP2;1 channel, Cd^2+^ interacts with negatively charged aspartate (D^28^) and glutamate (E^31^) residues located in the intracellular amino terminus, in a region suggested to anchor the loop D gating domain [49]; homologous residues are found in AtPIP2;1 (D^28^ and E^31^) and AtPIP2;2 (D^26^ and E^29^). Residues at E^31^, R^124^ and H^199^ in AtPIP2;1 also have been identified (based on site-directed mutagenesis to alanine) that resulted in changes in sensitivity of the AQP water channel function to divalent ions Ca^2+^ and Cd^2+^ [40]. The first known blocker of mammalian AQP1 water permeability was extracellularly applied Hg^2+^ which covalently binds to a cysteine (C^189^ in human AQP1) in the loop E domain, part of the extracellular water pore vestibule [50]. Mutation of this cysteine to serine or alanine had no effect on Cd^2+^-induced inhibition of ion currents in HsAQP1 expressing oocytes indicating that this cysteine is not involved in the reversible blocking effects of divalent cations described here. 

The HsAQP1, DmBIB, AtPIP2;1, and AtPIP2;2 channels showed voltage-sensitive inhibition by Ba^2+^ and Cd^2+^, and time- and voltage-dependent unblocking of the outward currents at positive potentials. The voltage-sensitivity of block suggests that the site of action of divalent cations for AQP cation channels is within the membrane electrical field. Although the observed outward rectification would be consistent with exit of the positively charged blocker towards the extracellular side of the channel during depolarization, the time required for onset of Cd^2+^ inhibition (over minutes) suggests that divalent ion permeation to a site on the intracellular side of the electrical field cannot be ruled out. DmBIB channels showed an increase in current amplitude after Ba^2+^ as compared with divalent-free saline, suggesting possible unblocking of the ion channel from a steady state level of inhibition perhaps involving an intracellular divalent cation. The competitive displacement of Ca^2+^ by excess Ba^2+^ (seen most dramatically in AtPIP2;1 and AtPIP2;2 ion channels) suggests that: (i) these divalent ions use the same site to inhibit function, (ii) the affinity for Ca^2+^ is greater than that for Ba^2+^, and (iii) the inhibitory effect is likely to be mediated by direct interaction rather than indirectly through Ca^2+^-dependent kinases. 

## 4. Materials and Methods

### 4.1. Preparation and Injection of Xenopus laevis Oocytes

The use of animals in this study was done in accord with the Guide for the Care and Use of Laboratory Animals, licensed under the South Australian Animal Welfare Act 1985, with protocols approved by the University of Adelaide Animal Ethics Committee (approval number M-2013-167, 20 September 2013). Oocytes were obtained from adult female *Xenopus laevis* frogs kept at the University of Adelaide Animal Laboratory Services. Oocytes were defoliculated using collagenase (type 1A, 1 mg/mL; Sigma-Aldrich, St. Louis, MO, USA) with trypsin inhibitor (0.05 mg/mL; Sigma-Aldrich) in calcium free saline containing 96 mM NaCl, 2 mM KCl, 5 mM MgCl_2_, penicillin 100 units/mL streptomycin 0.1 mg/mL, and 5 mM HEPES (4-(2-hydroxyethyl)-1-piperazineethanesulfonic acid) at pH 7.6, for 1 to 1.5 h. Oocytes were kept at 16 °C in standard Frog Ringers saline consisting of 96 mM NaCl, 2 mM KCl, 5 mM MgCl_2_, 0.6 mM CaCl_2_, 5 mM HEPES buffer, horse serum (5%; Sigma-Aldrich), penicillin 100 units/mL streptomycin 0.1 mg/mL, and tetracycline 0.5 mg/mL, pH 7.6. 

Aquaporin cDNAs were linearized and transcribed in vitro using published methods [9,11,13,51]. NCBI Protein Accession Numbers for the cDNA constructs used in the study are: AAA66221.1 (RnAQP5, *Rattus norvegicus*); AAC52152.1 (RnAQP4, *R. norvegicus*); NP_932766.1 (HsAQP1, *Homo sapiens*); NP_001030851.1 (AtPIP2;1, *Arabidopsis thaliana*); NP_181254.1 (AtPIP2;2, *A. thaliana*); NP_001190920.1 (AtPIP2;7, *A. thaliana*); P23645.2 (DmBIB, *Drosophila melanogaster*). The DmBIB cDNA sequence used here was modified as described previously to incorporate a hemagglutinin-epitope tag which did not impair channel expression or function [11]. 

Stage V–VI oocytes were selected and injected with 50 nL of sterile water (RNase free) containing 1 ng of HsAQP1, RnAQP4, or RnAQP5 cRNAs; 12 ng of AtPIP2;7, AtPIP2;2 or AtPIP2;1 cRNAs; or 20 ng of *Drosophila* BIB cRNA using a manual oocyte microinjection pipette (Drummond Scientific, Broomall, PA, USA). Oocytes injected with 50 nL of sterile water served as non-AQP-expressing (sham) controls. During the incubation post-injection to allow protein expression, oocytes injected with HsAQP1 or DmBIB cRNAs were maintained in standard Frog Ringers saline (2–5 days). Oocytes injected with AtPIP2;7, AtPIP2;2 or AtPIP2;1 cRNA were maintained in high potassium Frog Ringers saline (62 mM NaCl, 36 mM KCl, 5 mM MgCl_2_, 0.6 mM CaCl_2_, 5 mM HEPES buffer, horse serum 5%, penicillin 100 units/mL, streptomycin 0.1 mg/mL, and tetracycline 0.5 mg/mL, pH 7.6) for 1 to 1.5 days. Maintenance in high potassium Frog Ringers enhanced viability and extended the useful life of the AtPIP-expressing oocytes. Prior to experiments, all sham controls and AQP-expressing oocytes were rinsed in divalent-free saline for 10 min. To confirm successful expression, osmotic swelling assays were used for oocytes expressing AtPIP2;7, AtPIP2;2, AtPIP2;1, RnAQP4, or RnAQP5 or HsAQP1 channels, and by confocal immunostaining for oocytes expressing hemagglutinin-epitope tagged DmBIB channels, as per published methods [9,11,13,51].

### 4.2. Osmotic Swelling Assays

Control and AQP-expressing oocytes were rinsed in calcium free saline for 10 min. Swelling assays were performed in 50% hypotonic saline (calcium-free saline diluted with equal volume of water). Changes in the cross-sectional area of oocytes membranes were imaged using a grayscale camera device (Cohu, San Diego, CA, USA) mounted on a dissecting microscope (Olympus SZ-PT; Olympus, Macquarie Park, Australia). Images were captured at 0.5 per second for 60 s. Swelling responses of the oocytes were assessed with Image J software (National Institutes of Health, Bethesda, MD, USA) (Available online: http://rsbweb.nih.gov/ij/; accessed on 31 October 2017). Swelling rates were measured as slope values of the linear regression fits of relative volume as a function of time using Prism (GraphPad Software Inc., San Diego, CA, USA).

### 4.3. Two Electrode Voltage Clamp Recordings

Two-electrode voltage clamp recordings were used to record currents from AQP-expressing and control oocytes at room temperature. Capillary glass electrodes (1–3 MΩ) were filled with 1 M KCl. Recordings were performed in isotonic saline containing 100 mM NaCl, 2 mM KCl, 5 mM HEPES, and calculated amounts of CaCl_2_ buffered with 20 mM EGTA (ethylene glycol-bis(β-aminoethyl ether)-*N*,*N*,*N*′,*N*′-tetraacetic acid) to achieve the desired final concentration of free calcium, calculated using the online application (Available online: http://maxchelator.stanford.edu/CaEGTA-NIST.htm, accessed on 31 October 2017). The membrane permeable cyclic GMP analog, CPT-cGMP ((*R*p)-8-(*para*-chlorophenylthio)-cGMP; Sigma-Aldrich) was applied as a bolus into the extracellular bath to achieve a final concentration of 10 μM. Conductance responses were monitored through the experiments by repeated steps to +40mV (800-ms duration) every 6 s from a holding potential of −40 mV, using a GeneClamp amplifier and Clampex 9.0 software (pClamp 9.0 Molecular Devices, Sunnyvale, CA, USA). Data were filtered at 2 kHz and stored to a hard disk for analysis. Quantitative values of the magnitude of relative outward rectification were calculated by standardizing outward current amplitude (at +60 mV) to inward current amplitude (at −80 mV). The AQP1 ion channel blocker AqB011 was custom synthesized by Gary Flynn (Spacefill Enterprises, Bozeman, MT, USA) as described previously [19]. Statistical analyses were done with one-way ANOVA with Bonferroni post hoc tests; *p* values are indicated in the figure legends. 

### 4.4. Measurement of Oocyte Na^+^ Accumulation

The moles of Na^+^ in control and AtPIP2;2 expressing oocytes were measured three days after injection of sterile water or cRNA. Oocytes were incubated in Frog Ringers containing 96 mM NaCl. Five oocytes per sample were homogenized in 1 mL of 1% nitric acid and incubated at 75 °C for 1 h. Twenty control and 20 AtPIP2;2 expressing oocytes were included in the experiment, with five oocytes per sample and four replicate samples used. Aliquots were diluted in 1% nitric acid and the moles of Na^+^ in the oocytes were measured relative to known standards using a flame photometer (M410, Corning, NY, USA) following methods published previously [52].

### 4.5. Bath Application of Divalent Cations

The effects of divalent cations Ca^2+^, Mg^2+^, Ba^2+^ and Cd^2+^ were tested in oocytes expressing HsAQP1, AtPIP2;7, AtPIP2;2, AtPIP2,1 and DmBIB channels. Oocytes were placed in EGTA-buffered divalent-free saline for the initial electrophysiological recordings. Oocytes expressing HsAQP1 were treated with CPT-cGMP and monitored for 30 min to allow currents to reach maximal activation. Only 2 to 3 min were needed for oocytes expressing DmBIB, AtPIP2;1 and AtPIP2;2 to allow stabilization of the current response since these channels showed activation from the time the recording electrodes were placed in the oocyte membrane. After recording the initial activated responses, the bath saline was perfused with test salines containing Ca^2+^, Mg^2+^ or Ba^2+^ as described in the experiments; new steady state response levels were established within 1–2 min. The conductance responses were measured at 6 min steady state. Washout was done by perfusing the bath with divalent-free saline. 

## 5. Conclusions

Differential inhibition by divalent cations could be utilized by cells as a gating mechanism for adjusting AQP ion channel functionality in signal processing, volume control and fluid homeostasis processes. The regulation of AtPIP2;1, and AtPIP2;2 channels by Ca^2+^ is important for generating adaptive responses to environmental stressors [32]. AtPIP2;1 modulation by Ca^2+^ and pH may enable control of volume and turgor in guard cells and other cell types [53,54,55] but AtPIP2;1 is also permeable to H_2_O_2_, which is a component of signaling for guard cell closure [28]. AtPIP2;2 channels are abundantly expressed in roots, and essential for maintaining transmembrane water transport under osmotic stress conditions [27]. Non-Selective Cation Channels (NSCC) found in epidermal protoplast membranes of *Arabidopsis thaliana* roots are inhibited by Ca^2+^ and pH [41]; Byrt and colleagues [13] have hypothesized that AtPIP2;1 may be a molecular candidate for the identity of the NSCC channels. With the discovery here that AtPIP2;2 also can conduct ions, the number of molecular candidates for the NSCC is increasing.

Inhibition of ion channel function by AqB011 was confirmed for HsAQP1, but was not observed in oocytes expressing AtPIP2;1 or AtPIP2;2 channels. Based on in silico modelling, AqB011 previously was predicted to act at the intracellular side of the AQP1 channel at the loop D gating domain [19], specifically at a pair of arginine residues (the first two) within a series of four arginines that is highly conserved among vertebrate AQP1 channels, and also implicated in cGMP-mediated activation of the ionic conductance [10]. Interestingly, this pair of arginines is not present in AtPIP2;1 or AtPIP2;2 sequences, which instead have a proline residue as the first residue in the loop D series (Figure 7). These data are consistent with the idea that the loop D domain could be the target for the AqB011 interaction, and support the idea that AQP pharmacological agents can be expected to exhibit subtype selectivity based on differences in amino acid sequence in key functional domains of AQP channels. Understanding the pharmacological and physiological regulation of AQP ion channels is essential for defining their diverse array of functional properties and translational roles in living organisms across the kingdoms of life.
